# Hepatotoxic mechanism of cantharidin: insights and strategies for therapeutic intervention

**DOI:** 10.3389/fphar.2023.1201404

**Published:** 2023-06-13

**Authors:** Dian Jin, Na-Na Huang, Jing-Xia Wei

**Affiliations:** ^1^ Department of Pharmacy, Sixth People’s Hospital of Chengdu, Chengdu, China; ^2^ State Key Laboratory of Southwestern Chinese Medicine Resources, School of Pharmacy, Chengdu University of Traditional Chinese Medicine, Chengdu, China

**Keywords:** cantharidin, hepatotoxicity, autophagy, apoptosis, natural producct

## Abstract

Cantharidin (CTD), a natural compound derived from *Mylabris*, is widely used in traditional Oriental medicine for its potent anticancer properties. However, its clinical application is restricted due to its high toxicity, particularly towards the liver. This review provides a concise understanding of the hepatotoxic mechanisms of CTD and highlights novel therapeutic strategies to mitigate its toxicity while enhancing its anticancer efficacy. We systematically explore the molecular mechanisms underlying CTD-induced hepatotoxicity, focusing on the involvement of apoptotic and autophagic processes in hepatocyte injury. We further discuss the endogenous and exogenous pathways implicated in CTD-induced liver damage and potential therapeutic targets. This review also summarizes the structural modifications of CTD derivatives and their impact on anticancer activity. Additionally, we delve into the advancements in nanoparticle-based drug delivery systems that hold promise in overcoming the limitations of CTD derivatives. By offering valuable insights into the hepatotoxic mechanisms of CTD and outlining potential avenues for future research, this review contributes to the ongoing efforts to develop safer and more effective CTD-based therapies.

## 1 Introduction

Cancer continues to be the leading cause of death worldwide, significantly impacting human health, with an estimated 10 million fatalities expected in 2020 (Sung et al., 2021). Current clinical treatments for tumors, such as surgical resection, chemotherapy, and radiotherapy, often inflict considerable harm and pain on patients due to side effects (Nussbaumer et al., 2011). As a result, there is a pressing need for highly effective, targeted therapies with fewer side effects (Saad, 2022; Staudt et al., 2022; Zhang et al., 2022). Natural products have proven to be a valuable source for antitumor drug discovery, with approximately 50% of antitumor drugs in use today being derived directly or indirectly from plants, animals, and microorganisms (Harvey et al., 2015; Sznarkowska et al., 2017; Newman, 2020). Promising compounds for tumor treatment include alkaloids, flavonoids, terpenoids, polyphenols, quinones, and saponins. There has been growing interest in recent years in the use of toxic traditional Oriental medicine to treat malignant tumors. Long-term clinical practice in the Asia has shown that toxic traditional Oriental medicine, such as *Mylabris*, *Aconiti lateralis Radix Praeparata*, *Strychni semen*, and *Bufonis venenum*, possess significant antitumor effects. Active ingredients in these toxic compounds, including cantharidin (Wang et al., 2021), aconitine (Wang et al., 2020; Luan et al., 2022), and toadstool (Pan et al., 2019), exhibit unique pharmacology, offering promising therapeutic options for cancer treatment despite their toxicity.

*Mylabris* (Chinese: *Banmao*), a renowned TCM derived from the dried bodies of *Mylabris phalerata Pallas* or *Mylabris sichorii Linnaeus*, used for over 2000 years and is included in Sheng Nong’s Herbal Classic (Commission, 2020). CTD, a mono-terpene phosphoprotein phosphatase inhibitor, is the primary active ingredient of *Mylabris*, primarily used for the topical treatment of warts (Cotton, 2021; Guenthner et al., 2021). Recent studies have shown that CTD induces apoptosis in tumor cells, positioning it as a promising treatment for various malignancies, particularly hepatocellular carcinoma (Zeng et al., 2020). CTD demonstrates a unique advantage over first-line chemotherapeutic drugs by elevating leukocytes and cytokines, thus improving immune function (Wang, 1989; Florea and Büsselberg, 2011; Shou et al., 2013). CTD also exerts its antitumor effects by blocking the cell cycle, inducing apoptosis, and reversing multidrug resistance through various mechanisms (Zheng et al., 2008). Several *Mylabris*-/CTD-based drug formulations are available in the Chinese market, including *Aidi injection*, *disodium cantharidinate injection*, and *compound Mylabris capsules*, which have shown effective anti-tumor effects against liver cancer, lung cancer, rectal cancer, and malignant lymphoma (Liu et al., 2021; Wang et al., 2021; Wei et al., 2021; Yang et al., 2022). However, the therapeutic dose of CTD is very close to its toxic dose, with a lethal oral dose ranging from 10–60 mg and a median lethal dose (LD_50_) of 1.71 mg/kg in mice (Li et al., 2017).

Despite its potential as an antitumor agent (Shaoting et al., 2023), the low bioavailability, intestinal irritation, and significant hepatotoxicity of CTD limit its clinical application (Youyou et al., 2020). The current lack of a comprehensive understanding of CTD toxicity complicates effective clinical prevention and treatment. Consequently, developing an efficient delivery system for CTD or reducing its toxicity through structural modification may provide a solution. This review aims to summarize the hepatotoxicity mechanism of CTD and briefly introduce the progress in developing delivery systems for CTD and its derivatives, providing a reference for researchers and clinicians.

## 2 Hepatotoxicity of cantharidin

CTD poisoning has been associated with multi-organ damage, with acute circulatory failure and acute renal failure as leading causes of death in affected patients. Following oral administration, *Mylabris* initially stimulates the stomach, intestines, and other digestive organs, resulting in multi-organ damage with inflammation of the digestive tract, mucosal necrosis, and hepatocyte damage, including turbidity, steatosis, and necrosis (Bagatell et al., 1969; Zhang et al., 2020b; He et al., 2022). Toxic substances such as CTD in *Mylabris* can cause glomerular degeneration, tubular epithelial edema, and hemorrhage, leading to renal impairment and a significant increase in serum blood urea nitrogen and creatinine levels (Massicot et al., 2005; Cotovio et al., 2013). At the same time, toxic substances excreted from the kidneys can stimulate the urinary tract, ultimately causing symptoms such as urinary urgency, painful urination, and urinary abnormalities like hematuria and proteinuria. Stimulation of the urethra can also cause abnormal penile erection (Peng, 2014). Consequently, *Mylabris* is used as an aphrodisiac in some parts of the world but is deadly (Diaz et al., 2020). The absorption of toxic substances can directly damage capillary endothelial cells, leading to cell gap dilation and increased vascular permeability, resulting in the extravasation of plasma components. CTD can also cause turbid swelling of cardiomyocytes and myocardial hemorrhage (Knapp et al., 1997; Knapp et al., 1998; Zhang et al., 2020a).

**FIGURE 1:**
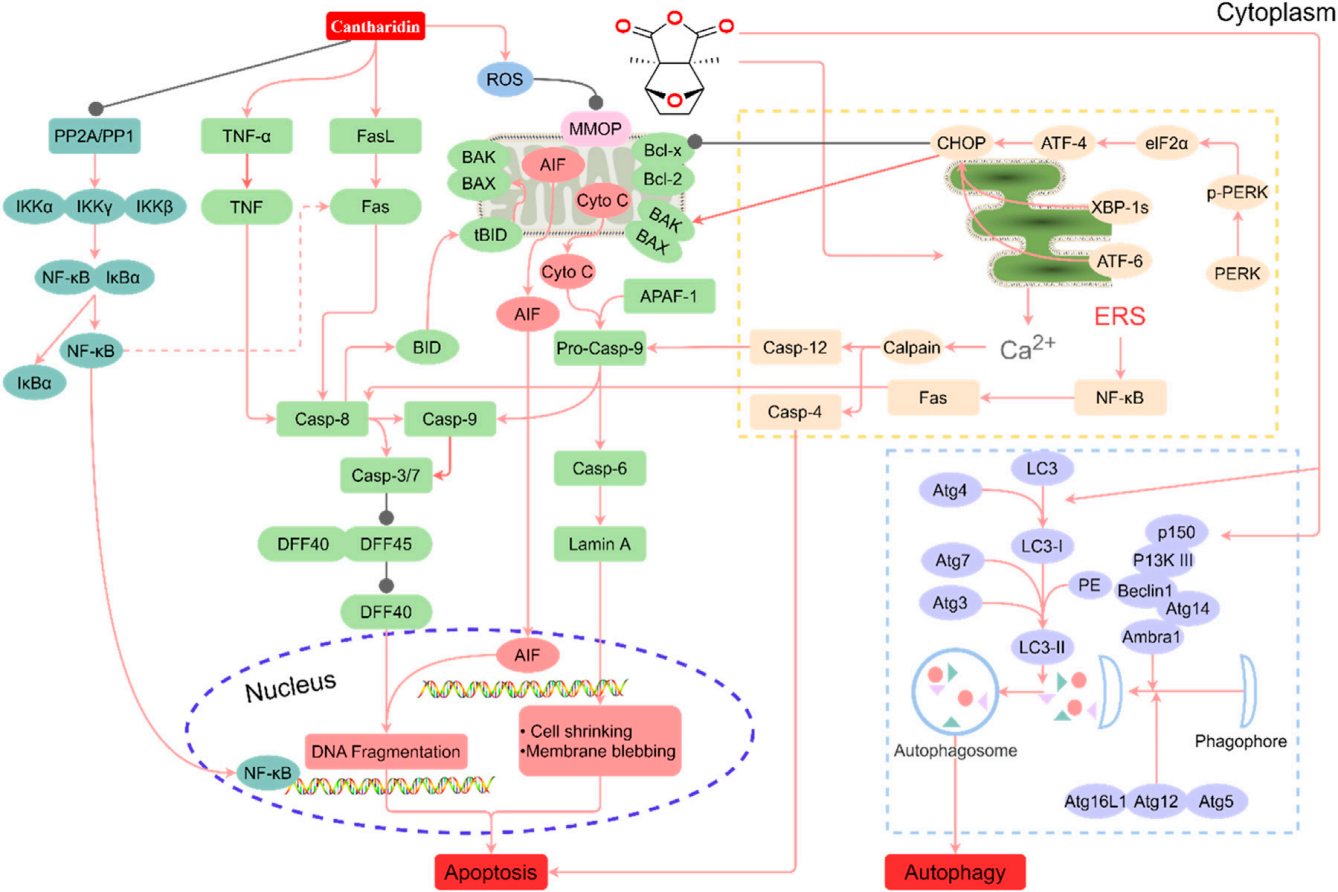
Summary of the mechanism of hepatotoxicity of cantharidin. In hepatocytes, cantharidin inhibits proliferation, promotes apoptosis and autophagy, and exacerbates the inflammatory response. These effects are associated with the inhibition of protein phosphatase (PP) 1, PP2A, Toll-like receptor (TIL)-4, nuclear factor-κB (NF-κB), ERK, and DFF45, and the promotion of the tumor necrosis factor (TNF)-α, FASL, ROS, caspase-4, caspase-6, caspase-8, caspase-9, caspase-12, protein kinase R-like ER kinase (PERK), inositol-requiring enzyme 1, transcription factor 6 (ATF6), BID, BAK, BAX, Cyto C, LC3-I, p150, Atg7, P13K Ⅲ, eIF2α, ATF-4, and CCAAT/enhancer-binding proteins-homologous protein (CHOP) pathways.

Various degrees of liver injury have been observed in reported cases of CTD poisoning or death (Zhang et al., 2018). As the primary organ involved in drug metabolism and detoxification, the liver is more vulnerable to drug damage than other organs (He et al., 2019). Studies have shown that the liver is the main target organ of CTD-induced toxicity (Wu et al., 2015; Zhang J. Y. et al., 2020; Liu et al., 2020c). Inflammatory cell infiltration, hepatocyte injury, degeneration, and necrosis are the primary pathological manifestations of CTD hepatotoxicity (Xu et al., 2013). Biochemical markers of liver injury, such as bilirubin, aspartate aminotransferase (AST), alanine aminotransferase (ALT), and alkaline phosphatase (ALP), were significantly upregulated in models with varying degrees of liver injury and cases of toxicity. CTD-mediated hepatotoxicity is mainly associated with endoplasmic reticulum stress (ERS), autophagy, activation of the cysteine signaling pathway, mitochondrial dysfunction, and bile acid cycle (Wu et al., 2015; Zhang J. Y. et al., 2020; Liu et al., 2020c; Liu et al., 2020; Yu et al., 2020) (Figure 1, Table 1).

**TABLE 1:** The mechanism of CTD’s hepatotoxicity.

Type of study	Cell lines and/or animal models	Concentration and duration	The specifific molecular mechanisms	References
*In vivo*	Male albino rats of the Simonson strain	10 mg/275 g for 35 min	Cell membrane, endoplasmic reticulum and mitochondrial damage in hepatocytes	Bagatell et al. (1969)
*In vivo*	Male Swiss-Webster mice	1 mg, 3 mg and 10 mg/kg for 30 min	-	Graziano et al. (1987)
*In vivo*	Mice	10 mg/kg	Liver enlargement and congestion; increased hepatic glycogenolysis	Graziano and Casida (1987)
*In vitro*	Partially purified PP2A (heterotrimeric complex ABC type) isolated from mouse brain	0.001, 0.01, 0.1, 1, 10, 100, 1,000 μΜ for 10 min	Inhibition of PP2A	McCluskey et al. (1996)
*In vitro*	Normal Chang liver cells; Hep 3B cells	0.5–25 μΜ for 36 h	Presence of lipid droplets; swelling of the mitochondria; accumulation of glycogen particles	Wang et al. (2000)
*In vivo*	Male Sprague–Dawley rats	0.5 mL/kg (cantharidinate injection) for 7 d	Inhibition of CYP2D6 and CYP3A4	Zhou et al. (2015)
*In vivo*	Kun Ming (KM) mice	1.5 mg/kg for 14 d	Deformed liver lobule and sinus hepaticus; deposited cytonecrosis, and inflammatory infiltration; elevated serum concentrations of glutamic-pyruvic transaminase (GPT) and glutamic oxaloacetic transaminase (GOT); upregulation of intrahepatic TNF-α, NF-κB, p-IκB, and toll-like receptor 4	Wu et al. (2015)
*In vivo*	Kun Ming (KM) mice; Human liver LO2 cells	0.5 mg/kg and 1.5 mg/kg for 14 days; 2 μΜ and 4 μΜ	Hepatocytes swelling, fine cytoplasmic vacuoles and necrosis; over-expression of caspase-3, caspase-8 and caspase-9 proteins in mice liver tissue and LO2 cells; increased levels of oxidized glutathione and/or glutathione in mice liver tissue	Zhu et al. (2019)
*In vitro*	Human liver LO2 cells	6.25 μΜ and 25 μΜ for 12 h	Elevated levels of ALT, AST and lactate dehydrogenase (LDH)	Liu et al. (2020c)
*In vitro*	Human liver LO2 cells	6.25, 12.5, 24, 50, and 100 μM for 24 h	Increase ALT, AST, LDH, and malondialdehyde levels; reduce glutathione peroxidase and superoxide dismutase activities; low concentrations of CTD induced the expressions of ERS-related proteins (GRP78, ATF4, PERK, p-PERK, XBP1–1s, and CHOP), but high concentrations of CTD inhibited their expressions; high concentrations of CTD activated autophagy (LC3, Beclin-1, Atg3, Atg4A, Atg4B, and Atg7), induced the expressions of apoptotic proteins (Bax/Bcl-2 and caspase-3)	Liu et al. (2020)
*In vivo*	Male Sprague-Dawley rats	1.34 mg/kg and 2.67 mg/kg 24 h	Elevated levels of TNF-α, IKK-α and caspase-3; raised IKK-α mRNA and caspase-3 mRNA levels; the ratio of Bcl-2/Bax increased in the low-dose group but decreased in the high-dose group	Yu et al. (2020)
*In vivo*	Sprague–Dawley rats	0.75 mg/kg and 1.5 mg/kg for 15 days	Increased liver weight; elevated liver index; sinusoidal cellularity was increased, the nuclei of the HCs were densely stained and varied in size, with the HCs exhibiting punctate necrosis, and vacuole change, partial cell edema, and disordered arrangement of HCs; increase ALT, AST, and total bilirubin (T-BIL) levels	Zhang J. Y. et al. (2020)
*In vivo*	Sprague–Dawley rats	1 mg/kg for 28 days	Increased levels of ALT, AST, and T-BIL; hyocholic acid (HCA), tauroursodeoxycholic acid (TUDCA) and tauro-β-muricholic acid (TβMCA) levels increased	Cheng W. et al. (2021)
*In vivo*	Male Sprague–Dawley rats	1 mg/kg for 14 days	Liver cell swelling or necrosis, and inflammatory cell infiltration, increase ALT, AST, ALP and LDH levels, upregulates the expression of lysoPE, lysoPC and triglyceride	Huang et al. (2021)

### 2.1 Endoplasmic reticulum stress

The endoplasmic reticulum (ER) is an essential organelle in hepatocytes, serving as the primary site of drug metabolism and responsible for proper folding and post-translational modification of membrane and secreted proteins (Wang and Kaufman, 2016). Unfavorable internal and external factors, such as drug-induced toxicity, hypoxia, and nutrient deprivation, can cause ER misfolding, unfolded protein accumulation, and calcium ion imbalance (Tabas and Ron, 2011). To counteract these disruptions, the ER initiates a signaling cascade called the ERS response, which aims to re-establish intracellular homeostasis and promote cell survival (Westrate et al., 2015). The ERS response activates through three transmembrane proteins: Protein kinase R-like ER kinase (PERK), inositol-requiring enzyme 1, and activating transcription factor 6 (ATF6). This response, known as the unfolded protein response, is triggered by the accumulation of misfolded or unfolded proteins and excessive calcium release due to membrane leakage (Di Conza and Ho, 2020). ERS and autophagy-related signaling pathways interact, playing a crucial role in acute liver injury. Under mild stress, hepatocytes activate ERS and autophagy mechanisms to protect cells from stress injury while inhibiting apoptosis. However, when stress levels increase and persist, apoptotic pathways are activated. Under severe stress, hepatocytes undergo complete necrosis, leading to tissue destruction (Liu et al., 2020).

A significant hallmark of ERS pathway-mediated apoptosis is the activation of CHOP, a member of the CCAAT/enhancer-binding proteins family encoded by the DDIT3 gene (Gu et al., 2017). CHOP can also act as a pro-apoptotic factor, promoting the expression of pro-apoptotic proteins such as BAK/BAX. Under normal physiological conditions, CHOP protein expression is extremely low. However, under various pathological conditions, including ERS, CHOP expression increases dramatically, activating apoptosis (Shah and Kumar, 2016; Yang et al., 2020). Liu et al. (2020) established an *in vitro* study model using LO2 cells and found that CTD mediates CHOP protein expression, leading to cell damage and apoptosis. Continuous treatment of LO2 cells with low concentrations of CTD activates the ATF6 and PERK pathways, initiating downstream signaling pathways and continuous accumulation of CHOP proteins, thereby inducing apoptosis. High concentrations of CTD (50 μM) inhibit ERS, promoting autophagy and apoptosis, and inducing toxicity in LO2 cells. Additionally, ERS causes an imbalance in calcium homeostasis, allowing calcium ions to escape from the ER to the cytoplasm, activating calpain and ultimately pro-apoptotic proteins caspase-4/7/12 (Kurokawa and Kornbluth, 2009; Hsia et al., 2014).

### 2.2 Autophagy

Autophagy is a conserved catabolic process in eukaryotic cells that maintains intracellular homeostasis and prevents various diseases (White, 2015). Although nutritional deficiencies typically activate autophagy, it is also associated with numerous physiological and pathological processes, including development, differentiation, neurodegenerative diseases, stress, infection, and cancer (Codogno et al., 2011). Induction of autophagy requires a class III PI3K complex containing hVps34, Beclin-1, p150, and Atg14-like proteins (Papinski and Kraft, 2014; Schneider and Cuervo, 2014). Atg genes regulate autophagosome formation through the Atg12-Atg5 and LC3-II complexes. Atg12 binds to Atg5 via a ubiquitin-like reaction requiring Atg7 and Atg10 (corresponding to E1 and E2-like enzymes, respectively). The Atg12-Atg5 complex non-covalently interacts with Atg16, forming a larger complex. Atg4 protease cleaves LC3/Atg8 at the carboxyl terminus, producing cytoplasmic LC3-I. LC3-I then binds to phosphatidylethanolamine through a ubiquitin-like reaction requiring Atg7 and Atg3 (corresponding to E1 and E2-like enzymes, respectively). The lipidated form of LC3, also known as LC3-II, attaches to the autophagosome membrane (Papinski and Kraft, 2014). Autophagy and apoptosis can be positively or negatively linked, with numerous interactions between the two processes. Bcl-2 inhibits Beclin-1-dependent autophagy, functioning as both a pro-survival and anti-autophagy regulatory molecule. Several pro-apoptotic signals, such as TNF, TRAIL, and FADD, can also induce autophagy. Liu et al. (2020) reported that treating LO2 cells with high concentrations of CTD *in vitro* significantly upregulated the expression of LC3, Beclin-1, Atg3, Atg4A, Atg4B, and Atg7, proteins associated with activated autophagy.

### 2.3 Caspase

Caspases, a group of cysteine proteases, play a critical and synergistic role in apoptotic signaling cascades. These enzymes can be activated via both exogenous and endogenous apoptotic pathways (Van Opdenbosch and Lamkanfi, 2019; Ketelut-Carneiro and Fitzgerald, 2022). Apoptosis is triggered by the activation of death receptors, such as Fas, TNFαR, DR3, DR4, and DR5, upon binding to their respective ligands. DNA damage, Ca^2+^ homeostatic imbalance, and ERS can also initiate apoptosis (Chao et al., 2022). Upon activation of pro-apoptotic factor receptors, caspases cleave and activate downstream effector caspases, including caspase-3, -6, and -7 (Swanton et al., 1999). Fas ligand binding results in Fas trimerization, which recruits the initiator caspase-8 through the adaptor protein FADD. Caspase-8 then undergoes oligomerization and autocatalytic activation (Pirnia et al., 2002). Subsequently, caspase-8 cleaves BID into truncated BID (tBID), which disrupts the outer mitochondrial membrane (Kaufmann et al., 2012). This disruption leads to the release of the pro-apoptotic factor cytochrome c (Cyto C), a crucial component for pro-caspase-9 activation (Kantari and Walczak, 2011). Cyto C, released from the membrane gap, binds to APAF1 (apoptosis protease activator-1), which recruits and activates caspase-9, ultimately leading to caspase-3 activation (Srinivasula et al., 1998; Pirnia et al., 2002). The activation of caspase-3 signifies the irreversible phase of apoptosis. During apoptosis, pro-apoptotic factors such as AIF, SMAC (mitochondrial-derived caspase activator), and DIABLO are released from the mitochondria alongside Cyto C. These factors promote caspase activation by inhibiting IAP (inhibitor of apoptosis) family proteins (Kaufmann et al., 2012). ERS induces Ca^2+^-mediated activation of caspase-12. TNF-α interaction with TNFαR activates the NF-κB pathways through NIK/IKKα/β/γ. The activation of NF-κB triggers the expression of pro-survival genes, including Bcl-2 and FLIP, which directly inhibit caspase-8 activation (Swanton et al., 1999; Pinkoski et al., 2000; Pirnia et al., 2002). According to Yu et al. (2020), male Sprague-Dawley rats exposed to low (1.34 mg/kg) and high (2.67 mg/kg) doses of CTD displayed increased expression levels of TNF-α protein and IKK-α genes as CTD doses increased. Wu et al. (2015) observed that CTD-induced chronic liver injury was associated with inflammatory cell infiltration and abnormal upregulation of TNF-α. Inhibition of the Toll-like receptor 4/NF-κB pathway attenuated CTD-induced hepatotoxicity.

### 2.4 Mitochondrial dysfunction

Mitochondria function as the center of cellular energy metabolism and play vital roles in cellular processes such as cell proliferation, genetic information transfer, immune regulation, cell cycle control, and apoptosis. Additionally, they are a major site for reactive oxygen species (ROS) production. Damage or dysfunction of mitochondria leads to metabolic abnormalities and functional organ decline in the body (Rustin et al., 1994), making it a potential contributor to liver toxicity. For example, in rats treated with CTD, liver mitochondria exhibited swelling and disappearance of cristae, potentially due to increased production of ROS free radicals causing organ dysfunction (Blajszczak and Bonini, 2017). Furthermore, the activation of BAK and BAX, two crucial apoptosis effectors, results in mitochondrial outer membrane permeabilization and Cyto C release (Bock and Tait, 2020).

### 2.5 Bile acid cycle

Glutathione plays a critical role in defending against oxidative damage and promoting integrated detoxification by scavenging nitrogen radicals and ROS, as well as reducing hydrogen peroxide. A decrease in glutathione levels is considered a potential early activation signal for apoptosis (Calabrese et al., 2017). Studies have demonstrated that CTD interferes with several biometabolic processes in mouse liver, causing significant disruptions in glutathione metabolism, taurine and hypotaurine metabolism, and the interconversion of pentose and glucuronide (Zhu et al., 2019). Specifically, oxidized glutathione, glutathione, 3-sulfoalanine, and deoxycholic acid 3-glucosylate are involved in three significantly disordered metabolic pathways (Zhu et al., 2019).

The bile acid cycle may play a crucial role in CTD-mediated hepatotoxicity, as disruption of BA homeostasis can lead to the accumulation of toxic BAs, resulting in cholestasis, bile duct infarction, liver fibrosis, and cirrhosis (Yang et al., 2017). Several studies have reported that BAs play an essential role in the hepatotoxicity of various drugs. For example, impaired BA homeostasis has been associated with milliporeline-induced hepatotoxicity and increased intracellular bile acid levels (Xiong et al., 2014). Taurine β-muricholic acid (TβMCA), taurocholic acid, and taurodeoxycholic acid (TDCA) are potential biomarkers of oleanolic acid-induced hepatotoxicity (Feng et al., 2020). Cheng W. et al. (2021) demonstrated that TβMCA levels significantly increased in rat liver following CTD (1.0 mg/kg) intervention. TβMCA is a competitive and reversible antagonist of the ligand-activated farnesoid X receptor, and elevated TβMCA levels can inhibit farnesoid X receptor activation and disrupt BA homeostasis. HCA, TUDCA, and TβMCA can serve as biomarkers for CTD-induced hepatotoxicity in rats (Cheng W. et al., 2021). However, the specific mechanisms underlying the roles of HCA, TUDCA, and TβMCA in CTD-induced hepatotoxicity warrant further investigation.

### 2.6 Other signaling pathway

In addition to causing severe hepatotoxicity, research on CTD poisoning has revealed that CTD can induce cardiotoxicity and nephrotoxicity (Zhang et al., 2018). CTD has been demonstrated to induce non-endothelium-dependent vasoconstriction in bovine coronary artery rings in a time- and concentration-dependent manner (Knapp et al., 1997; Knapp et al., 1998). Furthermore, exposure of erythrocytes to CTD results in erythrocyte shrinkage and membrane disorders, eventually leading to suicidal erythrocyte death (Alzoubi et al., 2015). *In vitro* exposure of HK-2 cells to CTD elevates levels of intracellular pro-apoptotic protein caspase-3 expression and the BAX/Bcl-2 ratio (He et al., 2020). CTD also activates the ERS-dependent PERK/CHOP pathway, inducing macroautophagy and apoptosis, which contributes to toxic effects on rat and HK-2 cells (He et al., 2022). Results from *in vivo* and *in vitro* experiments have shown that the expression levels of ERS regulatory genes, such as PERK, eIF2α, CHOP, and ATF4, are elevated alongside pro-apoptotic proteins, including GRP78, ATF4, LC3, Beclin-1, Atg3, Atg7, caspase-3, and the BAX/Bcl-2 ratio (He et al., 2022). Similar to CTD-mediated hepatotoxicity, CTD induces autophagy and apoptosis through ERS, leading to cardiotoxicity and nephrotoxicity.

## 3 Anticancer activity of cantharidin

Numerous clinical and experimental studies have evidenced the potent and wide-ranging antitumor properties of CTD on diverse cancer cell types. A comprehensive overview of these studies is provided in Table 2. These include investigations into cancers such as leukemia (Huh et al., 2004; Zhang et al., 2004; Dorn et al., 2009; Sun et al., 2016), bladder cancer (Huan et al., 2006; Kuo et al., 2010; Huang et al., 2013), rectal cancer (Huang et al., 2011; Han et al., 2014; Sheng et al., 2015), Ehrlich ascites cancer (Verma and Prasad, 2012; 2013), Dalton’s lymphoma (Prasad and Verma, 2013), oral cancer (Xi et al., 2015), pancreatic cancer (Wu et al., 2014; Xie et al., 2015), lung cancer (Hsia et al., 2014; Hsia et al., 2015a; Hsia et al., 2015b; Hsia et al., 2016), gastric cancer (Zhang et al., 2014), breast cancer (Zhang and Yan, 2015; Gu et al., 2017), renal cell carcinoma (Ren et al., 2016), skin cancer (Li et al., 2017), bile duct cancer (Zhou et al., 2018), and notably, liver cancer (Lu et al., 2014; Le et al., 2016; Zhang et al., 2017; Ma et al., 2018).

**TABLE 2:** Potential anticancer effects and related mechanisms of CTD.

Type of study	Cell lines and/or animal models	Concentration and duration and/or inhibition IC_50_ (μM)	Anticancer effects	References
*In vitro*	A2780, ADDP, 143B, HCT116 and HT29 cell lines	10 ± 2, 11 ± 1.2, 10 ± 1.2, 9 ± 1 and 6.4 ± 0.7 μM for 72 h	↓AA2P	McCluskey et al. (2000a)
*In vitro*	L1210, HL60, A2780, ADDP, 143B, HCT116, HT29, WiDr and SW480 cell lines	15 ± 2, 10 ± 2, 10 ± 2, 11 ± 1, 10 ± 1, 9 ± 1.1, 6.5 ± 0.5, 6.1 ± 0.5, 13 ± 5 and 7.8 ± 1 μM for 72 h	↑Cell cycle G2/M arrest, ↑apoptosis	Sakoff et al. (2002), McCluskey et al. (2003a)
*In vitro*	U937 cell lines	5, 10, 20 and 40 μM for 24 h	↓Cell viability, ↑p38, ↑caspase-3, ↑JNK MAP pathways	Huh et al. (2004)
*In vitro*	HL-60 cell lines	25 μM for 24 h	↓DNA replication, ↓DNA repair, ↓energy metabolism, ↑ATL-derived PMA-responsive peptide (Noxa), ↑Bcl-10, ↑TNF-α and ↑TGF-βIIR	Zhang et al. (2004)
*In vitro*	CCRF-CEM cell lines, lymphoblastoid TK6 cell lines with wild-type p53 and lymphoblastic WTK1 cells with a p53Ile273 mutation cell lines	0.625, 1.25, 2.5 and 5 μM for 24 h	↑Oxidative stress, ↑p53-dependent apoptosis, ↑DNA strand breaks, and ↑ROS	Efferth et al. (2005)
*In vitro*	T-24, RT4 and HT-29 cells	1.57, 3.13, 6.25, 12.5 and 25 μM for 24 h	↓Cell viability, ↑p38, ↑caspase-3, ↑caspase-7, ↑caspase-9, ↑Cell cycle G2/M arrest, ↑p21Cip1/Waf1, ↓PARP, ↓cyclin A/B1, ↓CDK1, ↑COX 2, ↑TNF-α, ↑PEG2, ↑phospho-eIF2α and ↑Grp78	Huan et al. (2006), Su et al. (2015)
*In vitro*	KB-3–1, MGC803, HepG3, HL-60 and Glc82 cell lines	2.7, 2.8 ± 0.6, 19.1, 2.7 and 1.2 μM for 72 h	↓Cell viability, ↑TNF-α and ↓ AA2P	Shan et al. (2006)
*In vitro*	HEK293T, LO2, HepG2/ADM and HepG2 cell lines	0.5, 1 and 2 μg/mL	↓MDR1, ↓P-glycoprotei and ↓mRNA transcription	Zheng et al. (2008)
*In vitro*	U266, RPMI-8226 and IM9 cells (Human myeloma cell lines)	5 μM for 24 h	↓JAK/STAT pathway, ↑apoptosis, ↓bcl-xL, ↑caspase-3, ↑caspase–8 and ↑caspase–9	Sagawa et al. (2008)
*In vitro*	HepG2, SK-Hep1 and Rat hepatocyte cell lines	11, 34 and 21 μM for 24 h	↓Cell viability	Yeh et al. (2010)
*In vitro*	PANC-1, CFPAC-1, BxPC-3 and Capan-1 cell lines	2, 4, 6, 8 and 10 μM for 24, 48 and 72 h	↓Cell viability, ↑apoptosis, ↑Oxidative stress, ↑cell cycle G2/M arrest, ↑TNF-α, ↑caspase-8 and ↑caspase-9	Li et al. (2010)
*In vitro*	TSGH 8301 cells	5, 10, 15, 20 and 25 μM for 24 h	↑Apoptosis, ↓mitochondrial membrane potential (*Δ*ψm), ↑cell cycle G0/G1 arrest, ↑caspase-3, ↑caspase-8, ↑caspase-9, ↑ROS, ↓cyclin E, ↓Cdc25c, ↑Endo G, ↑TNF-α, ↑AIF, ↑p21, ↑p-p53, ↓Bcl-2, ↑Bax and ↑PARP	Kuo et al. (2010)
*In vitro*	HeLa, ATCC CRL 5946, ATCC CRL 5915, ATCC CRL 1469 and ATCC CRL 1687 cell lines	2 and 5 μM for 24 h at pH 7.7 or 6.7	↓JAK/STAT pathway and ↑apoptosis	Fukamachi et al. (2010)
*In vitro*	Colo 205 cell lines	10, 20 and 40 µM for 24, 48 and 72 h	↑Cell cycle G2/M arrest, ↑ROS, ↑apoptosis, ↓CDK1, ↓Cyclin A, ↓Cyclin B, ↑CHK1, ↑p21 and ↓mitochondrial membrane potential (*Δ*ψm), ↓Bcl-2, ↑Fas/CD95, ↑caspase-3, ↑Cyto C and ↑Bax	Huang et al. (2011)
*In vitro*	PANC-1 and CFPAC-1cell lines	5, 10 and 20 µM for 24, 48 and 72 h	↓PP2A/IKKα/IκBα/p65 NF-κB pathway, ↓Wnt/β-catenin pathway, ↑TNF-α, ↑TRAILR1, ↑TRAILR2, ↑degradation of MMP2 mRNA, ↓ERK, ↓JNK, ↓PKC and ↓NF-κB	Li et al. (2011), Wu et al. (2014), Shen et al. (2015), Wang et al. (2015)
*In vivo*	Inbred Swiss albino mice by serial intraperitoneal (i.p.) transplantations of 1 × 106 viable EAC (Ehrlich ascites carcinoma) cells	0.5, 1, 1.5 and 2 mg/kg for 5 d	↓Proliferation, ↑apoptosis, ↑necrosis, ↑autophagy, ↑caspase-3/7, ↑caspase-9, ↓LDH activity	Verma and Prasad, 2012 (2013)
*In vitro*	MCF-7 cells	1.25, 2.5, 5, 10 and 20 µM for 24, 48 and 72 h	↑Apoptosis, ↓α2 integrin, ↓adhesion, ↓protein kinase C pathway and E2F1/MCM7-miR-106b-93/p21-PTEN signaling	Shou et al. (2013), Zhang and Yan (2015)
*In vivo*	Dalton’s lymphoma (DL) bearing mice	0.5 mg/kg for 5 d	↑Apoptosis, ↑necrosis, ↓mitochondrial membrane potential (*Δ*ψm), ↓glutathione, ↓succinate dehydrogenase activity, ↑caspase-3, ↑caspase-9 and ↑Cyto C	Prasad and Verma (2013)
*In vitro*	TSGH-8301 cells	1 and 2.5 µM for 24 and 48 h	↓Proliferation, ↑DNA damage, ↑apoptosis, ↓Matrix Metalloproteinase (MMP)-2/-9 Signaling, ↓p-p38 and ↓p-JNK1/2	Huang et al. (2013), Kuo et al. (2015)
*In vitro*	HCT-116, A549, PC-3and DU-145 cell lines	1, 5, 10 and 30 µM for 24 h	↑Apoptosis, ↓heat shock protein 70, ↓Bcl-2-associated Athanogene Domain 3, ↓Bcl-2, ↓Bcl-xL, and ↓Mcl-1	Kim et al. (2013)
*In vitro*	NCI-H460 cells	2.5, 5, 10, 15 and 30 µM for 48 h	↑DNA damage, ↑apoptosis, ↓4-3-3 proteins sigma (14-3-3r), ↓DNA-dependent serine/threonine protein kinase, ↓O6methylguanine-DNA methyltransferase and ↓mediator of DNA damage checkpoint protein 1	Hsia et al. (2015a), Hsia et al. (2015b)
*In vitro*	CHO-K1, UMSCC23, UMSCC10A and UMSCC10B cells	0–33 µM for 4, 8 and 12 h	↑ERS, ↑unfolded protein response	Xi et al. (2015)
*In vitro*	SGC-7901 and BGC-823 cells	2.5, 5, 10, 20, 40 and 80 µM for 24, 48 and 72 h	↑Cell cycle G2/M arrest, ↑caspase-7, ↑caspase-8, ↑caspase-9, ↑p21, ↓CDK1, ↓cyclin A, ↓cyclin B, ↓Bcl-2 and ↓Bid	Zhang et al. (2014)
*In vitro*	A375.S2 cells	1, 2, 3, 4 and 5 µM for 48 h	↑Cell cycle G2/M arrest, ↓Cdc25c, ↓Cyclin A, ↓PI3K/NF-ĸB Signaling Pathways, ↓ERK1/2, ↓PI3K, ↓FAK, ↓MMP-2, -9, ↓COX-2, ↓NF-ĸB p65, ↓TIMP 1, ↓TIMP 2, ↓VEFG, ↓uPA, ↓Rho A, ↓GRB2, ↓ROCK-1, ↓Ras, ↑p38, ↑JNK, ↑p-c-jun and ↑PKC	Hsiao et al. (2014), Ji et al. (2015), Mu and Sun (2018)
*In vitro*	H460 cells	5, 7.5, 10, 15 and 30 µM for 24 h	↓Cell viability, ↑ROS, ↑Ca2+-productions, ↓mitochondrial membrane potential (*Δ*ψm), ↑GRP78, ↑IRE1α, ↑IRE1β, ↑TNF-α, ↑ATF6α, ↑calpain 2, ↑XBP-1, ↓calpain 1, ↑Cyto C, ↑Bax, ↑caspase-3, ↑caspase-4, ↑caspase-8, ↑Cyto C, ↑Bax and ↑AIF	Hsia et al. (2014)
*In vitro*	QBC939 cells	2, 6 and 10 µM for 24 h	↑Apoptosis, ↑ROS and ↑IKKα/IκBα/NF-κB pathway	Zhou et al. (2018)
*In vitro*	TCA8113 cells	10, 20 and 40 µM for 48 h	↓Proliferation, ↑apoptosis, ↓miR-214, ↑p53, ↓Bcl2/Bax	Tian et al. (2015)
*In vitro*	MG-63, MNNG/HOS, U-2 OS, 143B and Saos-2 cells	0.5, 1, 2, 3 and 4 μg/mL for 24 and 48 h	↓Proliferation, ↑apoptosis, ↑Bax, ↓LEF1, ↑PARP, ↓Bcl-2, ↓p-Akt, ↓p-Cdc2, ↑DKK3, ↓miR-214-3p and ↓p-GSK-3β	Feng S. M. et al. (2018), Hu et al. (2021)
*In vitro*	HepG2, MHCC-97H, Hep3B, MHCC-97L, SMMC-7721 and Huh-7 cells		↓EphB4/PI3K/Akt signaling, ↓Bcl-2, ↓Mcl-1, ↑Bad, ↑Bax, ↑Bak, ↑TNF-α, ↑Cyto C, ↑caspase-7, ↑caspase −9 and ↑caspase −3	Zhu M. et al. (2020)

CTD primarily exerts its antitumor functions via multiple pathways, which include the inhibition of cell growth and proliferation, restriction of migration and invasion, along with the induction of apoptosis and autophagy. Succinctly, CTD obstructs the cell cycle, curbs cell migration, and triggers apoptosis in tumor cells through the regulation of an array of factors. These factors comprise apoptotic proteins (e.g., caspase-3/7/8, BAX, Bcl-2, Bcl-10, Fas/FasL, Beclin-1, Atg3/7), transcription factors (e.g., PPARα, NF-κB, Nrf2, STAT3), enzymes (e.g., AST, COX-2, SOD, eNOS), protein kinases (e.g., ERK, JAK2, p38, p53, P13K/Akt, mTOR), growth factors (e.g., TGF-β1, VEGAF, PDGF), and inflammatory cytokines (e.g., TNF-α, IL-1, IL-6, MCP-1). For a more in-depth exploration of the specific signaling pathways involved, a review of the antitumor effects of norcantharidin (a derivative of CTD) by Zhou et al. (2020) is recommended.

## 4 Potential solutions to cantharidin-mediated toxicity

**FIGURE 2:**
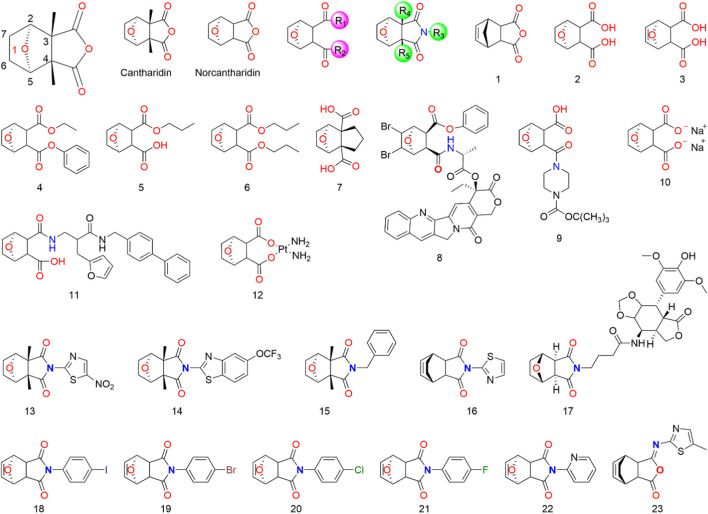
Structure of CTD and its derivatives.

Given the potent anticancer properties of CTD, alongside its severe side effects, it is crucial to develop approaches that reduce toxicity while preserving its activity. Although CTD demonstrates potent inhibition of protein phosphatase 2A and cytotoxic activity in cancer cells, its preclinical development might be hindered by its toxicity. To tackle this issue, chemists have synthesized various CTD derivatives (Figure 2) and developed several nano-precision delivery systems, some of which have shown promising antitumor potential.

### 4.1 Structural modification of cantharidin

Cantharidin is a natural protein phosphatase monoterpene anhydride inhibitor (Ren and Kinghorn, 2021), primarily composed of a six-membered carbon ring with an oxygen bridge and a five-membered anhydride ring. The activity of CTD is highly dependent on retaining the 1-O group (McCluskey et al., 1996; McCluskey et al., 2003b; Baba et al., 2005; Dong et al., 2007; Deng et al., 2013). Although the 1-O of CTD can be replaced by N, S, or -CH_2_- using the isostere principle, structural modification at the 1-position may significantly reduce or even eliminate the pharmacological activity of CTD derivatives. It is speculated that the 1-O can form a hydrogen bond with the receptor, facilitating proper binding between CTD and the receptor. Consequently, CTD derivatives with other atoms or groups replacing the oxygen atom might not bind effectively to the receptor, resulting in a loss of activity (Dong et al., 2007). Studies suggest that eliminating the bridging ether oxygen on the ring can decrease cytotoxicity (Yeh et al., 2010). Moreover, the presence of 2-C and 5-C substituents eliminates the inhibitory activity of CTD derivatives on all protein phosphatase 2A, even those with minimal steric hindrance (McCluskey et al., 1996; McCluskey et al., 2000b). Substitutions at 3-C and 4-C also significantly impact the toxicity and efficacy of CTD. For instance, norcantharidin, a derivative of CTD with comparable anti-tumor efficacy but fewer side effects than CTD, is primarily used for hepatocellular carcinoma treatment. It retains functions of raising leukocytes, protecting liver cells, and regulating immune function (Zhou et al., 2020). Substitutions at 6-C and 7-C increase the molecule’s spatial resistance, leading to decreased activity or selectivity. The transformation into carbon-carbon double bonds is an effective means of modification (Tatlock et al., 1997; Dong et al., 2007).

Modifying the CTD anhydride site has proven effective in enhancing its anti-hepatocarcinogenic activity. Cantharidic acid, a binary carboxylic acid formed by the ring-opening of CTD’s five-membered carboxylic anhydride ring, exhibits the same inhibitory activity against PPA as CTD (McCluskey et al., 2000b). Carboxylate derivatives of CTD are significant modifications, and sodium cantharidinate, which has been clinically applied, has a substantially superior antitumor effect compared to CTD, with lower toxicity and irritation (McCluskey et al., 2000b; Feng I. C. et al., 2018; Ji and He, 2019). The inhibitory activities of carboxylate derivatives of CTD against PPA1, PPA2A, and PPA2B vary depending on their structures. It has been reported that an amination reaction at the CTD anhydride site or the introduction of basic groups into the structure can effectively improve its anti-hepatocarcinoma activity (Dong et al., 2007; Wang et al., 2018; Zhou et al., 2020). The activity of imide derivatives varies significantly depending on the substituents attached to the nitrogen atom.

### 4.2 Cantharidin targeted delivery system

In clinical practice, CTD is typically administered at a dose range of 0.5–4 mg/d (Dang and Zhu, 2013). To address issues related to membrane irritation, *in vivo* release control, and limited bioavailability, it is essential to develop an effective drug delivery system. Nanoparticle-based drug delivery systems show promise in overcoming the limitations of conventional anticancer drug therapy. Sheng et al. developed folic acid-targeted nanoparticles loaded with CTD, which effectively killed colorectal cancer cells via a PP2A-dependent mechanism (Sheng et al., 2015). Chi et al. (2019) reported on norcantharidin-conjugated carboxymethyl chitosan conjugates for hepatocellular carcinoma treatment, which significantly inhibited the proliferation and migration of BEL-7402 cells. Dang and Zhu (2013) developed CTD-loaded solid lipid nanoparticles (CA-SLNs) with an oral bioavailability and sustained release profile after oral administration. Xie et al. (2021) designed a TriPt prodrug, combining cisplatin, artesunate, and CTD in equimolar ratios. TriPt NPs exhibited substantial antitumor effects in 7404DDP tumor-bearing mice and significantly improved drug efficacy compared to free drug combination therapy. Sun et al. (2021) developed a hepatic-targeting hyaluronic acid-mPEG-modified CTD nanostructured liposome (HA-mPEG-CTD-NLC) that inhibited hepatoma carcinoma cell growth and prolonged survival in tumor-bearing mice. Zhu K. et al. (2020) developed a novel CTD-loaded nanoliposome 18-GA-Suc-CTD-Lip, modified with 3-succinyl-30-stearyl glycyrrhetinic acid, which showed high toxicity against hepatocellular carcinoma cells. Cheng X. et al. (2021) developed a CTD-loaded biomimetic metal-organic framework nanoparticle cascade, PPy-CTD@MIL-100@MPCM nanoparticles (PCMM NPs), which accumulated in tumor tissue through encapsulated macrophage cell membranes (MPCMs) targeting inflamed tissue. This study suggested that PCMM NPs could serve as a combined treatment platform to enhance the Fenton reaction-based amplified photothermal therapy. Finally, Guo et al. (2020) developed a cell membrane-coated biomimetic nanoparticle (m-CTD@Te) with strong homologous targeting capabilities that effectively suppressed cancer through synergistic treatment. Encapsulated Te in m-CTD@Te triggered PDT and PPT by NIR laser irradiation, and PTT further triggered the release of CTD. Due to the outer cell membrane coating of m-CTD@Te, these nanoparticles exhibited good biocompatibility with healthy cells.

In brief, the advanced delivery systems discussed above have shown potential to reduce the toxicity of CTD and their derivatives by precise delivery to target cells, avoiding the potential side effects (Table 3). However, these systems do not fully meet the high clinical requirements needed. Although these systems can improve the therapeutic of CTD and its derivatives compared to direct utilization of CTD, their delivery efficiency, cell and tissue selectivity, and the physicochemical properties of the compounds delivered can significantly impact the therapeutic effects of CTD (Li et al., 2017). Hence, further research efforts are necessary to investigate these factors to improve the therapeutic efficacy and safety of CTD.

**TABLE 3:** Summary of CTD and its derivatives targeted delivery systems.

Dosage form	Drugs	Excipients	Advantage	References
Nanoparticles	CTD	Folic acid	Targeting, low toxicity	Sheng et al. (2015)
Conjugates	Norcantharidin	Carboxymethyl chitosan	Hepatic-targeting, long retention time in the blood circulation, low cardiac and renal toxicity	Chi et al. (2019)
Solid lipid nanoparticles	CTD	Glyceryl monostearate	Highly orally bioavailable, hepatic-targeting, slow-release *in vivo*, low toxicity	Dang and Zhu (2013)
Nanoparticles	CTD, cisplatin, artesunate	Methoxy poly (ethylene glycol)_5000_-*b*-poly (lactide-co-glycolide)_7600_ (mPEG_5000_-PLGA_7600_)	Synergistic effect, targeting, biosecurity, low toxicity	Xie et al. (2021)
Nano-lipid carrier	CTD	Hyaluronic acid (HA)-decorated copolymer (mPEG-NH_2_), hyaluronic acid	Tumor-targeting, slow-release *in vivo*, high bioavailability, low toxicity	Sun et al. (2021)
Nano-lipid carrier	CTD	3-succinyl-30-stearyl glycyrrhetinic acid	Hepatic-targeting, biosecurity, high bioavailability	Zhu K. et al. (2020)
Nanoparticles	Norcantharidin	Galactosylated chitosan	Hepatic-targeting, sustained and pH-sensitive release, strong cytotoxicity against hepatocellular carcinoma cells	Wang et al. (2010)
Nanoparticles	Norcantharidin	Soybean phosphatidylcholine	Hepatic-targeting, pH-sensitive, high bioavailability	Qiao-Ling et al. (2012)
Metal-organic framework nanoparticle	CTD	Polypyrrole, macrophage cell membranes, FeCl_3_·6H_2_O, Polyvinylpyrrolidone, MIL-100	Photothermal therapy, hepatic-targeting, pH-sensitive	Cheng W. et al. (2021)
Biomimetic nanoparticles	CTD	Polyvinylpyrrolidone, Na_2_TeO_3_	Photothermal therapy, homologous targeting, good biocompatibility	Guo et al. (2020)

## 5 The clinical usage of cantharidin

Although CTD has some toxicity to humans, its anticancer effects should not be overlooked. To reduce these side effects, several CTD derivatives, such as norcantharidin, disodium cantharidinate, and methylcantharidinmide, have been produced. These derivatives retain the antitumor effects of CTD, while reducing its toxic side effects and providing application advantages. Currently, National Medical Products Administration of China has approved several antitumor chemicals based on these CTD derivatives and several antitumor proprietary Chinese medicines containing *Mylabris* for the treatment of various solid tumors, particularly liver cancer. Table 4 provides a summary of the names, dosage forms, compositions, indications, specifications, and usage of these marketed preparations in China. However, it is vital to acknowledge that while such marketed products, which include CTD as one of the therapeutic components, have shown positive therapeutic outcomes in clinical settings, these beneficial effects may be attributed to other molecules contained within the products. Therefore, further elucidation of the exact pharmacological impacts of individual molecules is crucial to enhance future clinical application and usage guidelines.

**TABLE 4:** Marketed products containing *Mylabris*/Cantharidin or its related bioactive ingredients.

Marketed product	Drug dosage form	Composition	Indications	Specification	Usage	References
Aidi Injection	Injection	Mylabris, Panax ginseng, Astragali Radix, Acanthopanax senticosus	For primary liver cancer, lung cancer, rectal cancer, etc.	10 mL each	Intravenous drip	An et al. (2022)
Delisheng Injection	Injection	Mylabris, Red ginseng, Astragali Radix, Bufonis Venenum	For middle and advanced primary liver cancer with *Qi* deficiency and blood stasis syndrome	10 mL each	Intravenous drip	Dong et al. (2014)
Demethylcantharidin Tablets	Tablet	Norcantharidin	For hepatocellular carcinoma, esophageal cancer, gastric cancer, cardia cancer, leukopenia, hepatitis, cirrhosis, and hepatitis B virus carriers	5–15 mg	Oral	Zhou et al. (2020)
Ganning Tablets	Tablet	Mylabris, Arnebiae Radix, Glutinous rice	For treating a variety of acute and chronic hepatitis, especially abnormal liver function and hepatitis B patients with positive surface antigen, can prevent hepatitis B cancer	Each tablet weighs 0.3 g	Oral	Deng et al. (2011)
Disodium Cantharidinate Injection	Injection	Disodium Cantharidinate	For primary liver cancer, etc.	0.1 mg/2 mL	Intravenous drip	Fan (2010)
				0.25 mg/5 mL		
				0.5 mg/10 mL		
Disdium Cantharidinate and Vitamin B6 Injection	Injection	Disodium Cantharidinate and Vitamin B6	For liver cancer, lung cancer and leukopenia. It can also be used for hepatitis, cirrhosis and hepatitis B virus carriers	0.05 mg/5 mL	Intravenous drip	Zhu K. et al. (2020)
				0.1 mg/10 mL		
Compound Cantharidin Capsule	Capsule	Mylabris, Panax ginseng	For primary liver cancer, lung cancer, rectal cancer, malignant lymphoma, gynecological malignant tumor, etc.	0.25 g/capsule	Oral	He et al. (2021)
		Astragali Radix, Acanthopanax senticosus, Sparganii Rhizoma, Scutellaria barbata, Curcuma zedoaria, Corni Fructus, Ligustri Lucidi Fructus				
		Bear bile powder, licorice, etc.				

## 6 Conclusion

Despite the outstanding efficacy of CTD and high demand, Chinese patent medicines made of *Mylabris* or CTD, such as *Aidi injection*, *disodium cantharidinate injection*, and *compound Mylabris capsules*, have excellent efficacy in treating malignant tumors like liver cancer, breast cancer, leukemia, and other difficult-to-treat diseases. However, the direct use of CTD causes strong irritation to the skin and gastrointestinal mucosa, as well as significant damage to major organs like the liver, kidney, and heart, especially significant hepatotoxicity. Notably, drug-related liver injury is a leading cause of drug development interruption or marketed drug withdrawal, making it important to increase studies on the toxicity of CTD and its derivatives beyond hepatotoxicity to further enhance their clinical applications (Kaplowitz, 2005; Navarro and Senior, 2006; Hebels et al., 2014).

In this review, we summarized the mechanism by which CTD induces hepatotoxicity, leading to different degrees of liver injury through the activation of endogenous and exogenous pathways, resulting in apoptosis and autophagy in hepatocytes. Future research should focus on understanding the toxic reactions of CTD (Hong et al., 2022), studying the mechanism of CTD toxicity in-depth, and developing methods to reduce toxicity and improve the efficacy of CTD analogs based on the mechanism of CTD toxic reactions. Additionally, exploiting the unique advantage of CTD to enhance leukocytes among many antitumor drugs and increasing the use of CTD analogs alone or in combination with other antitumor drugs is a promising approach (Swati and Raghuvir, 2022). Furthermore, the development of nano-precision delivery systems to control the side effects of CTDs and enhance their targeting of tumor sites presents an exciting avenue for future research. By offering valuable insights into the hepatotoxic mechanisms of CTD and outlining potential avenues for future research, this review contributes to the ongoing efforts to develop safer and more effective cantharidin-based therapies.

In addition to these advancements, the development of targeted protein degradation technology has revolutionized the study of traditional small molecule compounds (Lin et al., 2022). PROTAC molecules, composed of E3 ubiquitin ligase ligand, protein of interest, and linker, have shown potential to break through existing applications when using natural products as protein of interest (Dhanusha and Craig, 2020). Therefore, the use of CTD and its derivatives, or other toxic compounds from traditional oriental drugs, as potential protein of interest, could enhance the therapeutic potential of CTD (Miaomiao et al., 2022). Although no results have been reported yet, this strategy deserves attention. In addition to targeted nano-delivery systems, antibody-drug conjugate technology offers a promising avenue by combining “specific” targeting and “efficient” killing of cancer cells (Anish et al., 2016). These drugs act like precision-guided “biological missiles” that can destroy cancer cells with precision, increase the therapeutic window, and reduce off-target side effects (Carmen et al., 2021). Research in this area may provide a significant breakthrough in the clinical use of CTDs, but further experimental validation is necessary.
